# Do Patients Accurately Recall Their Preoperative Symptoms After Elective Orthopedic Procedures?

**DOI:** 10.7759/cureus.36810

**Published:** 2023-03-28

**Authors:** Saad Masud, Joshua D Piche, Aditya Muralidharan, Ahmad Nassr, Ilyas Aleem

**Affiliations:** 1 Department of Orthopaedic Surgery, Wayne State University School of Medicine, Detroit, USA; 2 Department of Orthopaedic Surgery, University of Michigan, Ann Arbor, USA; 3 Department of Orthopaedic Surgery, Mayo Clinic, Rochester, USA

**Keywords:** orthopedics, patient-reported outcome measures, recall bias, patient recall, surgery

## Abstract

Patient-reported outcome measures are a frequent tool used to assess orthopedic surgical outcomes. However, recall bias is a potential limitation of these tools when used retrospectively, as they rely on patients to accurately recall their preoperative symptoms.

A database search of Cochrane Library, PubMed, Medline Ovid, and Scopus until May 2021 was completed in duplicate by two reviewers. Studies considered eligible for inclusion were those which reported on patient recall bias associated with orthopedic surgery. The primary outcome of interest investigated was the accuracy of patient recollection of preoperative health status. Any factors that were identified as affecting patient recall were secondary outcomes of interest.

Of the 4,065 studies initially screened, 20 studies with 3,454 patients were included in the final analysis. Overall, there were 2,371 (69%) knee and hip patients, 422 (12%) shoulder patients, 370 (11%) spine patients, 208 (6%) other upper extremity patients, and 83 (2%) foot and ankle patients. Out of the eight studies that evaluated patient recall within three months postoperatively, seven studies concluded that patient recall is accurate. Out of the 13 studies that evaluated patient recall beyond three months postoperatively, nine studies concluded that patient recall is inaccurate.

The accuracy of patient recall of preoperative symptoms after elective orthopedic procedures is not reliable beyond three months postoperatively.

## Introduction and background

Relieving pain, improving quality of life, and restoring function to the body are some of the common goals in orthopedic surgery. When assessing the success of procedures, patient-reported outcome measures (PROMs) are frequently relied on by both surgeons and researchers as validated tools that can be used to help guide patient care and shared decision-making [1]. Analyses of patient quality of life, pain, or disease-specific outcome measures are some of the more common PROMs that are collected [2]. The subjective nature of patients reporting their outcomes has limitations, but an even larger issue can present when they are asked to retrospectively recall their preoperative health or symptoms, as this type of assessment inherently can introduce recall bias [3].

Studies such as case series, case controls, or other retrospective designs do not typically include the prospective collection of preoperative information, and, therefore, often rely on patients to accurately recall their preoperative health status [4]. This is inherently problematic because assessing outcomes using personal recall can introduce recall bias, which has been previously described as a potential source of systemic error [5]. Many patient-related factors can influence the effects and strength of recall bias, including the duration of symptoms, the severity of preoperative health or pain, and current health. A comprehensive understanding of recall bias and the factors that influence it is critical when attempting to draw conclusions from orthopedic studies using retrospective PROMs [3].

Although many previous clinical studies have evaluated recall bias within orthopedic surgery, to our knowledge, there are no systematic studies to date that attempt to synthesize the effects of recall bias after orthopedic surgery procedures [3,6,7]. Given the prevalence of the use of PROMs within orthopedic surgery, this systematic review was performed to provide a review of the accuracy of patients’ recollection of their preoperative status after elective orthopedic surgical procedures. This information can help better guide researchers and clinicians when evaluating data from and designing future studies using PROMs.

## Review

Methods

**Figure 1:**
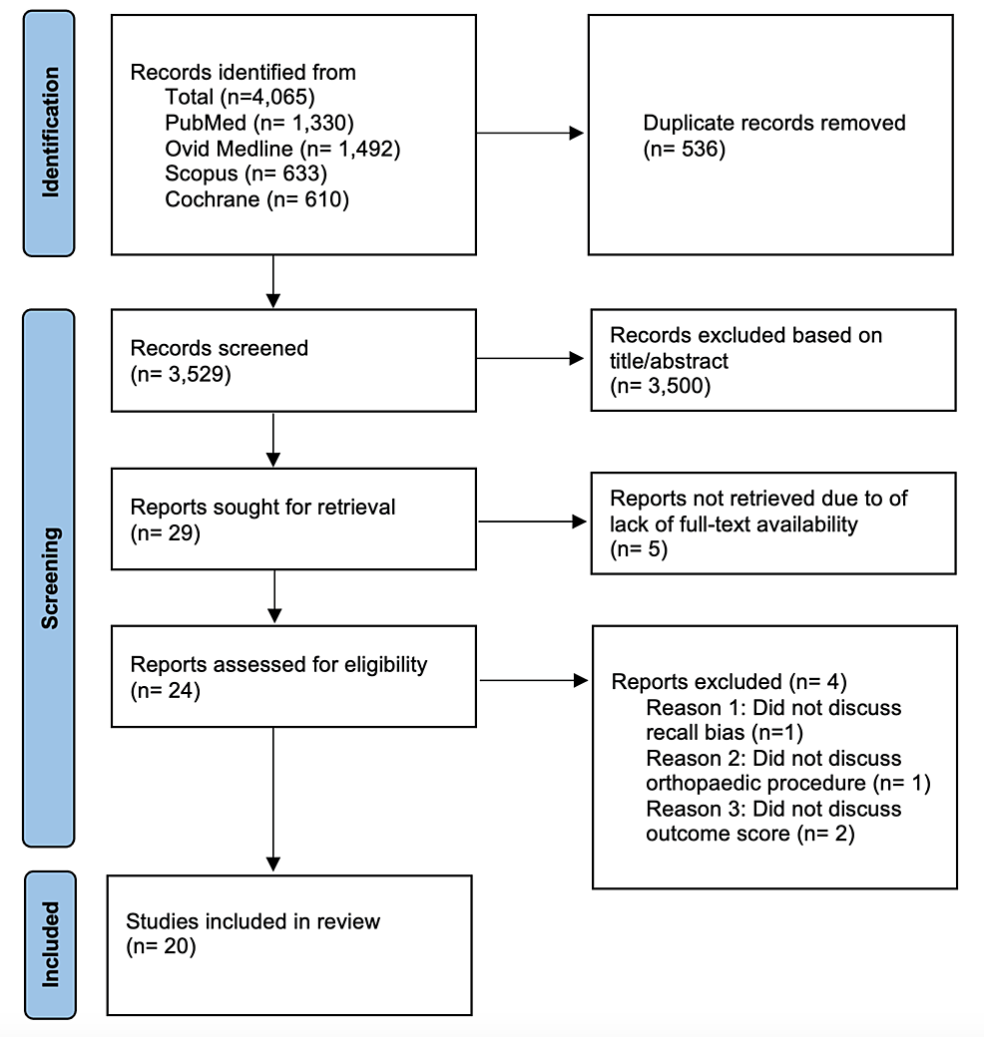
Preferred Reporting Items for Systematic Reviews and Meta-Analyses (PRISMA) flow diagram of article selection. n: number of studies

Using the search terms and keywords seen in Appendices, two reviewers (SM, JP) in duplicate performed searches using EMBASE, Cochrane, OVID Medline, and PubMed databases from all time points up until May 2021. This systematic review utilized both the Cochrane Handbook of Systematic Reviews and the Preferred Reporting Items for Systematic Reviews and Meta-Analyses (PRISMA) guidelines to ensure the quality and reproducibility of the process [8,9]. The entire search process is outlined in Figure 1. The two-reviewer process began with a screening of all titles and abstracts produced through the initial search of the four databases. Titles and abstracts that were deemed potentially relevant then underwent a thorough full-text analysis by both reviewers, constituting the second stage of review. At the end of this process, all titles included in the final analysis were agreed upon by the duplicate reviewers as well as the senior author. The senior author of the study provided a final consensus on any title in the case of any disagreement between reviewers.

To be considered for inclusion in this systematic review, studies must have used an adult (age of 18 years or older) cohort, be written in the English language, and must have specifically reported on the accuracy of patient recall of preoperative symptoms or quality of life after an orthopedic surgery procedure. The primary outcome of interest was the accuracy of patient recall of their preoperative symptoms or quality of life after they underwent an orthopedic surgery procedure. Secondary outcomes of interest included the accuracy of patient recall stratified by the duration of time between surgery and when they were asked to recall their preoperative symptoms.

The Critical Appraisal Skills Programme (CASP) and the Methodological Index for Non-Randomized Studies (MINORS) tools were used to grade and ensure the quality of included randomized and non-randomized studies, respectively [10-12]. This stage of the review, as well as all others which were performed in duplicate, utilized the methods of Landis et al. to ensure appropriate inter-rater reliability [13].

In addition to collecting the baseline demographics of all studies included in this systematic review, additional pertinent information was harvested including the type of orthopedic surgery performed, the tool or tools used for assessing the patient’s quality of life or symptoms preoperatively and postoperatively, and the duration of time between surgery and when patients were asked to recall their preoperative symptoms or quality of life. Lastly, all results pertaining to the accuracy of patient recall of their preoperative symptoms or quality of life were recorded.

Results

Study Identification

After the search terms were applied in all four databases, a total of 4,065 titles/abstracts were produced for the initial stage of review. Of these, 536 were deemed to be duplicates, and once these were removed, there were 3,529 studies with unique titles. Once these titles and abstracts were assessed, 24 studies were deemed relevant for a full-text review. The full-text review was performed, and this resulted in 20 studies with a total of 3,454 patients, which met the inclusion criteria and were deemed eligible to be included in this analysis. Table 1 shows the demographics of the 20 included studies. The comprehensive results pertaining to patient recall bias after orthopedic surgery can be seen in Table 2 and Table 3. A Cohen’s kappa (k) coefficient >0.8 between reviewers existed during all stages of the review, confirming strong interobserver reliability.

**Table 1:** Basic information and demographics of included studies. N: sample size; %: percentage; ACL: anterior cruciate ligament; MINORS: Methodological Index for Non-Randomized Studies; CASP: Critical Appraisal Skills Programme

Author	Year	Type of study	N	Mean age at surgery	% female	Procedure(s) evaluated	Quality assessment tool	Quality assessment score
Aleem et al. [6]	2016	Prospective cohort	62	66.1	58%	1. Lumbar decompression. 2. Lumbar fusion. 3. Lumbar decompression and fusion	MINORS	14
Aleem et al. [7]	2018	Prospective cohort	73	58.2	52%	1. Cervical decompression. 2. cervical decompression and instrumented fusion	MINORS	14
Bryant et al. [14]	2006	Randomized clinical trial	344	37.5	38%	1. Knee arthroscopy. 2. Knee arthroscopy with ACL repair	CASP	Acceptable
Finsen et al. [28]	2018	Prospective cohort	75	52	57%	1. Carpal tunnel release. 2. Thumb basal joint arthrosis surgery. 3. Hardware/Foreign body removal. 4. Other hand surgery	MINORS	11
Gotlin et al. [22]	2020	Prospective cohort	84	57.4	-	1. Arthroscopic rotator cuff repair	MINORS	13
Hillesund et al. [29]	2018	Prospective cohort	133	53	53%	1. Subacromial impingement surgery. 2. Carpal tunnel release. 3. Thumb basal joint arthrosis surgery. 4. Hardware/Foreign body removal. 5. Other hand surgery	MINORS	11
Hope et al. [23]	2019	Prospective cohort	119	24.5	14.3%	1. Arthroscopic anterior labral repair. 2. Arthroscopic posterior labral repair. 3. Arthroscopic anterior and posterior labral repair. 4. Open Latarjet	MINORS (comparative score)	19
Howell et al. [15]	2008	Prospective cohort	104	61	55%	1. Total hip arthroplasty	MINORS	11
Kwong et al. [16]	2018	Prospective cohort	443	69.1	65%	1. Total hip arthroplasty. 2. Total knee arthroplasty	MINORS	13
Lawson et al. [17]	2020	Prospective cohort	88	68	51%	1. Total Hip arthroplasty. 2. Total knee arthroplasty	MINORS	13
Lingard et al. [18]	2001	Prospective cohort	770	70	59%	1. Total knee arthroplasty	MINORS	11
Lowe et al. [24]	2016	Prospective cohort	169	67.6	54%	1. Anatomic total shoulder arthroplasty. 2. Reverse total shoulder arthroplasty. 3. Revision total shoulder arthroplasty	MINORS	11
Marsh et al. [19]	2009	Randomized clinical trial	174	70.6	50.6%	1. Total hip arthroplasty	CASP	Acceptable
Murphy et al. [20]	2015	Prospective cohort	113	58.2	52%	1. Total hip arthroplasty. 2. Total knee arthroplasty	MINORS	11
Pellisé et al. [26]	2005	Prospective cohort	58	48.3	52%	1. Posterior lumbar fusion with instrumentation	MINORS	8
Rodrigues et al. [27]	2018	Prospective cohort	177	54	58.6%	1. Surgery for degenerative cervical spine disease. 2. Surgery for degenerative lumbar spine disease	MINORS	11
Toolan et al. [30]	2001	Prospective cohort	47	-	-	1. Elective ankle surgery. 2. Elective hindfoot surgery	MINORS	10
Widnall et al. [31]	2014	Prospective cohort	36	54.6	52%	1. Forefoot surgery. 2. Hindfoot surgery	MINORS	10
Wilson et al. [25]	2009	Prospective cohort	50	57	52%	1. Total shoulder arthroplasty. 2. Arthroscopic subacromial decompression. 3. Acromioclavicular joint excision. 4. Rotator cuff surgery	MINORS	13
Yeoman et al. [21]	2018	Prospective cohort	335	72.5	53.7%	1. Total hip arthroplasty. 2. Total knee arthroplasty	MINORS	11

**Table 2:** Main results of included studies 1-10. PROM: Patient-reported outcome measure; NPS: Numeric Pain Score; ODI: Oswestry Disability Index; NDI: Neck Disability Index; WOMET: Western Ontario Meniscal Evaluation Tool; ACL-QOL: Anterior Cruciate Ligament Quality of Life; IKDC: International Knee Documentation Committee; KOOS: Knee Injury and Osteoarthritis Outcome Score; SF: Short-Form Health Survey; PRWHE: Patient-Rated Wrist/Hand Evaluation; ASES: American Shoulder and Elbow Surgeons; QuickDASH: Shortened Disability of the Arm Shoulder and Hand; MISS: Melbourne Instability Shoulder Score; WOSI: Western Ontario Shoulder Instability Index; WOMAC: Western Ontario and McMaster Universities Osteoarthritis Index; EQ-5D-3L: EuroQol 5 Dimension 3 Level; VAS: Visual Analogue Scale; *: statistically significant difference

Author & year	Follow-up time	PROMs used	Mean preoperative scores	Mean recalled scores	Difference between scores	Can patients accurately recall preoperative status?
Aleem et al., 2016 [6]	1 year	NPS Back	5.2	7.6	+2.3 (p < 0.05)*	No
		NPS Leg	5.0	6.8	+1.8 (p < 0.05)*	
		ODI	40.9%	50.3%	+9.6% (p < 0.05)*	
Aleem et al., 2018 [7]	14.5 months	NPS Neck	4.4	5.9	+1.5 (p < 0.05)*	No
		NPS Arm	4.1	6.4	+2.3 (p < 0.05)*	
		NDI	35.0%	40.8%	+5.8% (p < 0.05)*	
Bryant et al., 2006 [14]	2 weeks	WOMET	35.8	34.6	-1.12 (p = 0.21)	Yes
		ACL-QOL	33.4	36.6	+3.24 (p = 0.01)*	
		IKDC	45.5	45.6	+0.03 (p = 0.96)	
		KOOS	57.4	57.9	+0.50 (p = 0.33)	
		SF-36 Physical	39.9	39.0	-0.90 (p = 0.06)	
		SF-36 Mental	52.1	52.6	+0.43 (p = 0.49)	
Finsen et al., 2018 [28]	21 months	PRWHE (Carpal Tunnel)	47	50	+3	Yes – Group; No – Individually
		PRWHE (Thumb Arthrosis)	64	66	+2	
		PRWHE (Other)	26	51	+25	
Gotlin et al., 2020 [22]	39.1 months	ASES	51.42	30.69	-20.72 (p < 0.01)*	No
Hillesund et al., 2018 [29]	21 months	QuickDASH	-	-	+10	Yes – Group; No - Individually
Hope et al., 2019 [23]	7 months	MISS	48.81	47.76	-1.05 (p = 0.6)	Yes – Group; No – Individually
		WOSI	1250.28	1288.91	+38.64 (p = 0.34)	
	20 months	MISS	46.08	46.33	+0.25 (p = 0.91)	
		WOSI	1241.00	1265.02	+24.02 (p = 0.63)	
Howell et al., 2008 [15]	3 days	WOMAC Global	-	-	-3.08 (p < 0.05)*	Yes
		WOMAC Pain	-	-	-2.53 (p < 0.05)*	
		WOMAC Stiffness	-	-	-2.21	
		WOMAC Function	-	-	-4.64 (p < 0.01)*	
		Oxford Hip	-	-	+1.58 (p < 0.01)*	
		SF-12 Physical	-	-	+0.34	
		SF-12 Mental	-	-	-4.82 (p < 0.01)*	
	6 weeks	WOMAC Global	-	-	-1.46	
		WOMAC Pain	-	-	-1.34	
		WOMAC Stiffness	-	-	+3.23	
		WOMAC Function	-	-	-1.97	
		Oxford Hip	-	-	+0.85	
		SF-12 Physical	-	-	+0.23	
		SF-12 Mental	-	-	-2.79 (p < 0.01)*	
	3 months	WOMAC Global	-	-	-1.14	
		WOMAC Pain	-	-	-1.60	
		WOMAC Stiffness	-	-	+0.99	
		WOMAC Function	-	-	-0.76	
		Oxford Hip	-	-	+0.02	
		SF-12 Physical	-	-	-0.05	
		SF-12 Mental	-	-	+0.24	
Kwong et al., 2018 [16]	30 days	Oxford Hip	15.07	14.56	-0.51 (p = 0.15)	Yes
		Oxford Knee	17.36	17.02	-0.34 (p = 0.29	
		EQ-5D-3L Hip	0.24	0.22	-0.02 (p = 0.30)	
		EQ-5D-3L Knee	0.35	0.32	-0.03 (p = 0.16)	
Lawson et al., 2020 [17]	15 days	EQ-5D-5L Index	0.4691	0.4470	-0.02	Yes – Group; No – Individually
		EQ-VAS	66.89	66.36	-0.53	

**Table 3:** Main results of included studies 11-20. PROM: Patient-reported outcome measure; WOMAC: Western Ontario and McMaster Universities Osteoarthritis Index; SF: Short-Form Health Survey; VAS: Visual Analogue Scale; PROM: Patient reported outcome measure; ASES: American Shoulder and Elbow Surgeons; LEFS: Lower Extremity Functional Scale; FT: Feeling Thermometer; WDI: Waddle Disability Index; EQ-5D: EuroQol 5 Dimension; COMI: Core Outcome Measures Index; Numeric Rating Scale; NDI: Neck Disability Index; ODI: Oswestry Disability Index;  AOFAS: American Orthopaedic Foot and Ankle Society; FFI: Foot Function Index; *: statistically significant difference

Author & year	Follow-up time	PROMs used	Mean preoperative scores	Mean recalled scores	Difference between scores	Can patients accurately recall preoperative status?
Lingard et al., 2001 [18]	3 months	WOMAC	-	-	61% differed by more than 10%	No
		SF-36	-	-	50% differed by more than 10%	
Lowe et al., 2016 [24]	6 weeks	VAS Pain	6.2	7.0	+0.9 (p = 0.21)	Yes – Up to 6 weeks; No – After 6 weeks
		ASES Function	14.1	13.7	-0.4	
		ASES Total	32.8	28.7	-4.2 (p = 0.39)	
	3 months	VAS Pain	6.7	7.9	+1.1 (p < 0.01)*	
		ASES Function	13.0	12.2	+0.2	
		ASES Total	29.5	22.4	-5.4 (p < 0.01)*	
	6 months	VAS Pain	6.5	7.6	+1.1 (p < 0.01)*	
		ASES Function	13.7	13.5	-0.2	
		ASES Total	31.3	25.7	-5.6 (p < 0.05)*	
	12 months	VAS Pain	6.2	7.5	+1.3 (p < 0.01)*	
		ASES Function	14.4	12.8	-1.6	
		ASES Total	34.3	25.3	-9.0 (p < 0.01)*	
Marsh et al., 2009 [19]	6 weeks	LEFS	12.9	13.82	+0.92 (p = 0.19)	Yes
		WOMAC	49.31	49.27	-0.04 (p = 0.96)	
		Oxford Hip	39.42	42.16	+2.74 (p < 0.05)*	
		SF-12 Physical	25.03	27.85	+2.83 (p < 0.01)*	
		SF-12 Mental	53.16	51.12	-2.04 (p = 0.10)	
		FT	58.16	53.11	-5.06 (p < 0.01)*	
Murphy et al., 2015 [20]	12.4 months	Oxford Hip	20.17	19.37	-0.8 (p = 0.329)	No
		Oxford Knee	19.56	19.67	+0.11 (p = 0.912)	
Pellisé et al., 2005 [26]	37.5 months	Prolo	5.12	4.43	-0.69 (p < 0.01)*	No
		Pain-Prolo	2.07	1.60	-0.47 (p < 0.01)*	
		Function-Prolo	3.05	1.03	-2.02 (p = 0.0573)	
		WDI	52.36	70.83	+18.47 (p < 0.01)*	
		VAS	6.96	8.16	+1.2 (p < 0.01)*	
Rodrigues et al., 2018 [27]	14.1 months	EQ-5D	0.288	0.068	0.22 (p < 0.05)*	Yes – Cervical surgery; No – Lumbar surgery
		Health-VAS	50	30	-20.0 (p < 0.05)*	
		COMI Neck	7.4	7.9	+0.5	
		Neck NRS	6	7	+1.0	
		Arm/Shoulder NRS	6	7	+1.0	
		NDI	44	48	+4 (p < 0.05)*	
		COMI Back	7.9	9.0	+1.1 (p < 0.05)*	
		Back NRS	7	8	+1.0 (p < 0.05)*	
		Leg/Buttock NRS	8	9	+1.0 (p < 0.05)*	
		ODI	52	64	+8.0 (p < 0.05)*	
Toolan et al., 2001 [30]	> 6 months	AOFAS	-	-	-5.3 (p < 0.05)*	No
Widnall et al., 2014 [31]	137 days	FFI	103.1	135.1	+32 (p < 0.01)*	No
		SF-12 Physical	37.4	33.6	-3.8 (p < 0.01)*	
		SF-12 Mental	49.4	47.0	-2.4 (p < 0.01)*	
Wilson et al., 2009 [25]	50 days	Oxford Shoulder	39.4	40.5	+1.1 (p < 0.05)*	Yes – Group; No – Individually
Yeoman et al., 2018 [21]	1 year	Oxford Hip	20.9	20.9	-0.04 (p = 0.97)	Yes – Group; No – Individually
		Oxford Knee	21.85	20.26	-1.59 (p = 0.10)	

Overall Results

All 20 included studies reported on the accuracy of patient recall of preoperative status using at least one validated PROM following an orthopedic surgical procedure. In total, there were 2,371 (69%) knee and hip patients, 422 (12%) shoulder patients, 370 (11%) spine patients, 208 (6%) hand and elbow patients, and 83 (2%) foot and ankle patients across all 20 included studies. The primary outcome of interest was the accuracy of patient recall of preoperative health status. Among the 20 included studies, eight agreed retrospective collection of data was inaccurate, six found it inaccurate in individuals but not in large groups, four concluded it was accurate, one observed it to be inaccurate beyond six weeks, and one determined it to be accurate within spine surgery among patients who underwent surgery for cervical degenerative disease.

Recall Bias in Knee and Hip Surgery

Bryant et al. analyzed 344 patients who underwent arthroscopic knee surgery with or without anterior cruciate ligament repair [14]. The authors observed that although recalled data did have greater associated variances and patients were unable to accurately recall specific experiences, they showed good recall of average experiences. This study concluded that after two weeks postoperatively, patients undergoing knee surgery can accurately recall their preoperative quality of life, general health, and functional status.

Howell et al. evaluated 104 patients who underwent a total hip replacement [15]. This study found that patients’ age did influence recall ability, as patients over 65 had a weaker correlation between preoperative and recalled scores at three days postoperatively when compared to patients younger than 65 years. The authors concluded that patients can accurately recall their preoperative function for up to three months after total hip arthroplasty; however, not all scores were equally recalled with the Oxford Hip and Western Ontario and McMaster Universities Arthritis Index (WOMAC) scores being particularly reliable, and the 12-Item Short Form Health Survey (SF-12) score being the least reliable.

Kwong et al. reported on 443 patients who underwent either total hip replacement (n = 204) or total knee replacement (n = 239) surgery [16]. One observation was that recall was worse among patients over 75 when compared to patients under 60 for the Oxford Hip score. The study concluded that PROMs can be collected retrospectively to derive a baseline health status when it is not feasible or cost-effective to collect them prospectively.

Lawson et al. identified 88 patients who underwent total hip replacement (n = 29) or total knee replacement (n = 59) surgery [17]. They concluded that health-related quality of life outcomes measured retrospectively are almost equivalent to those measured prospectively at a group level but not at an individual level.

Lingard et al. examined 770 patients who underwent total knee replacement surgery [18]. It was noted that patients who had a worse three-month postoperative WOMAC function score, who were older than 75, or who had poorer mental health (36-Item Short Form Health Survey (SF-36) mental health score <60 points) had a significantly poorer recall of function. The authors concluded that retrospectively recalling preoperative status is not as accurate as collecting them prospectively when determining a patient’s change in symptoms or health status after an intervention, and at best only substitutes for quantifying symptoms before and after surgery.

Marsh et al. investigated 174 patients who underwent total hip replacement surgery [19]. The authors commented that agreement between actual and recalled data was excellent for disease-specific questionnaires and moderate for generic health measures; however, there were greater variances related to recalled data. This study concluded that preoperative general health, quality of life, and function can be recalled at six weeks postoperatively in patients undergoing total hip replacement with adequate accuracy to substitute the prospective collection of baseline data.

Murphy et al. discussed outcomes for 113 patients who underwent either total hip replacement (n = 59) or total knee replacement (n = 54) surgery [20]. While no significant differences were found between the actual and recalled preoperative scores, there were comparatively large absolute differences (Oxford Hip, 5.24; Oxford Knee, 5.41) and a weak correlation. Furthermore, for individual questions, the agreement between actual and recalled health status was poor for half of the Oxford Hip and two-thirds of the Oxford Knee scores. This study concluded that in patients undergoing total knee or hip replacement, the recollection of preoperative pain and function is inaccurate one year after surgery.

Yeoman et al. analyzed 335 patients who underwent either total hip replacement (n = 178) or total knee replacement (n = 157) surgery [21]. The authors concluded that when evaluating a group of patients, recalled preoperative scores can be substituted for prospective data collection for up to one year following hip and knee replacement. However, when evaluating individual patients, recalled preoperative scores cannot be depended upon because of poor reliability at one year post-procedure.

Recall Bias in Shoulder Surgery

Gotlin et al. evaluated 84 patients who were arthroscopically operated on for rotator cuff repair [22]. One observation was that patients who had less severe shoulder dysfunction preoperatively had a greater difference between preoperative and recall American Shoulder and Elbow Surgeons (ASES) scores (p < 0.001). It was also observed that older age was associated with an inaccurate recall of ASES scores (p = 0.062). It was concluded that recalled PROMs are susceptible to significant recall bias as they were almost always lower than their equivalent prospectively collected scores.

Hope et al. reported on 119 patients who underwent arthroscopic surgery for labral repair [23]. It was noted that there was no significant difference between the mean recalled scores and the actual mean group scores collected preoperatively. The conclusion drawn from this paper was that while mean recalled scores can accurately be used to obtain a baseline for a group, individual recall of preoperative conditions, even within a younger group of patients, is too inaccurate to be used for research.

Lowe et al. identified 169 patients who underwent total shoulder replacement surgery [24]. The authors concluded that preoperative function could be recalled accurately through the ASES function score for up to 12 months after their surgery; however, patients recall having worse pain six weeks postoperatively, thus making ASES total scores unreliable when they are recalled.

Wilson et al. examined 50 patients who underwent shoulder surgery, including total shoulder replacement, subacromial decompression, acromioclavicular joint excision, and rotator cuff procedures [25]. The authors noted that older age did not have an adverse effect on recall of preoperative shoulder symptoms. Additionally, recalled scores were noted to have a 95% chance of falling within an 18-point spread of the actual preoperative score which is much greater than 4.5 points, which is considered clinically significant. It was concluded that individual patient recall of preoperative symptoms is poor and unreliable; however, when considering a large group, a retrospective collection of preoperative status is not subject to recall bias.

Recall Bias in Spine Surgery

Aleem et al. examined 62 patients who received lumbar decompression and fusion surgery [6]. An interesting observation made was that over 40% of patients had changed their predominant symptom at recall from back pain to leg pain or vice versa. This study concluded that patient recollection of preoperative symptoms is inaccurate in lumbar spine surgery.

Aleem et al. discussed outcomes for 73 patients who underwent cervical decompression and fusion surgery [7]. The authors observed that over 30% of patients (44.4% short-term, 28.2% long-term) had switched their chief complaint from neck to arm pain or vice versa at recall. This study concluded that patients cannot accurately recall their preoperative status after cervical spine surgery.

Pellisé et al. analyzed 58 patients who underwent lumbar fusion surgery [26]. The authors noted that aside from the Prolo function subscale, there was a significant difference when comparing prospective and recalled preoperative data, with retrospective collection always demonstrating a worse recall of preoperative status. They concluded that when treating low back pain, reliance on patient recall of preoperative status is an ineffective method to establish a baseline status and can lead to overestimating the effectiveness of surgery.

Rodriguez et al. evaluated 177 patients who underwent surgery for degenerative cervical or lumbar spinal diseases [27]. It was observed that a sizeable portion of lumbar patients recalled worse scores compared to their preoperatively collected scores, and, therefore, overestimated the effect of surgery. Recall bias was worse for back pain and disability. Furthermore, within the lumbar patients, those who had self-assessed the surgery as being helpful were also noted to recall significantly worse scores compared to patients who classified the surgery as not helpful and recalled similar median scores for all PROMs. This study concluded that while a retrospective collection of preoperative status may be acceptable for patients with cervical degenerative diseases, in patients with lumbar degenerative diseases, it is inaccurate and may lead to an overestimation of the effectiveness of surgery, highlighting the importance of collecting data prospectively and not retrospectively when assessing the outcomes of spinal surgery.

Recall Bias in Hand and Elbow Surgery

Finsen et al. reported on 75 patients who underwent hand surgery for carpal tunnel syndrome (n = 26), thumb basal joint arthrosis (n = 9), hardware/foreign body removal (n = 4), and a variety of other conditions (n = 36) [28]. The authors noted that if 10 is subtracted from the mean recalled preoperative score for a group of patients, the real preoperative score will fall within ±4 of this score with a 95% confidence interval (CI). It was concluded that while recalled scores may be acceptable to evaluate the baseline of a group of patients, they are far too inaccurate to be useful in individual patients.

Hillesund et al. identified 133 patients who received hand surgery for various conditions including subacromial impingement (n = 29), carpal tunnel syndrome (n = 27), thumb basal joint arthrosis (n = 15), hardware/foreign body removal (n = 10), and others (n = 52) [29]. One observation from this study was that when 9 was subtracted from the recalled score, the mean difference from the real preoperative score will fall within ±4 of this score with a 95% CI. The authors concluded that remembered preoperative scores in individual patients are too inaccurate to be useful, even when corrected for; however, for a group of 30 or more patients, retrospective collection of preoperative status can be utilized with useful accuracy when corrected for in patients who have been surgically treated for carpal tunnel syndrome, subacromial impingement, or thumb basal joint arthrosis.

Recall Bias in Foot and Ankle Surgery

Toolan et al. examined 47 patients who underwent elective ankle or hindfoot surgery [30]. It was observed that patients who were dissatisfied with their surgical outcome recalled higher preoperative scores compared to their actual recorded scores. The conclusion drawn from this study is that recalled preoperative scores after elective foot and ankle surgery are a poor predictor of a patient’s actual preoperative status and can lead to an overestimation of the benefit of surgery.

Widnall et al. investigated 36 patients who were electively operated on with either forefoot (n = 21) or hindfoot (n = 15) surgery [31]. The authors observed that while retrospective scoring lacks accuracy compared to prospective scoring, data collected through the SF-12 score is significantly more accurate than the Foot Function Index score (p < 0.001), with data being recalled to within 10% of the actual preoperative score. This is likely due to the fewer elements present within the SF-12 decreasing the opportunity for error. Despite this, they concluded that, overall, patients tend to recall their preoperative status at a worse level than their true condition collected preoperatively, especially those who underwent forefoot procedures.

Discussion

This systematic review of the literature identified 20 studies that assessed the accuracy of patient recall of preoperative symptoms after undergoing orthopedic surgery. A major trend that this review found is that patient recall of preoperative symptoms or quality of life seems to be impacted in large part by the duration of the period when they are asked to recall the symptoms postoperatively because the accuracy of recall tended to be poorer as patients were further out from surgery. Of the eight studies that assessed patient recall within three months postoperatively, four studies concluded patients could accurately recall their preoperative status, and two studies concluded that at a group level, patients could accurately recall their preoperative status, but individually could not. One study found that up to six weeks patients could accurately recall their preoperative function, but past this, they could not. Only one study found that patients could not accurately recall preoperative status at three months postoperatively.

When looking at the 13 studies that evaluated patient recall of preoperative symptoms or health quality more than three months postoperatively, eight studies found that patients could not accurately recall their symptoms. There were four studies that found that at the group level, patient recall was accurate; however, at the individual level, patients could not accurately recall their preoperative symptoms. Lastly, one study found that patient recall at 14 months after cervical spine surgery was accurate; however, recall was not accurate after lumbar spine surgery.

Age appeared to be an additional factor identified, which affected patient recall accuracy, with four studies specifically addressing this. One study reported that those over 65 had poorer recall than those younger than 65 [15]. Another study reported that those over 75 had worse recall than those younger than age 60 [16]. One study found that those over 75 tended to have poorer recall of preoperative symptoms [18]. Finally, one study concluded that older age correlated with worse recall scores [22].

Outside of orthopedic surgery, other medical fields have also investigated the effects of patient recall bias on reported outcomes. For example, Flynn et al. assessed 30-day patient-reported recall of urinary tract infection symptoms in 254 patients [32]. They found that recall bias significantly affected answers on nine items that were tested, among 25% of the study population. They also reported that patient-related factors, such as depression or anxiety, and the severity of disease were associated with the overreporting of symptoms at the time of recall. Findings such as these highlight the likely multifactorial and complex phenomenon of patient recall bias.

While the effect of recall bias in medicine can affect PROMs, surgeons must also keep in mind that recall bias can affect them as well. Alsubaie et al. performed a study that assessed surgeons’, fellows’, and medical students’ abilities to recall major events from cases only seven to nine days after the case occurred [33]. The authors reported that nearly universally, all participants were unable to accurately recall the events.

Strengths and limitations

One major strength of this study is that it is the first report in the literature to systematically review and synthesize the effects of patient recall bias in the field of orthopedic surgery. This thorough review can aid providers when they are considering using PROMs to evaluate the outcomes of their patients following surgery. It can also help clinical researchers when they are tasked with designing a study that uses PROMs.

A major limitation of this review is that the 20 studies included were heterogeneous in terms of the type of surgery performed, the level of surgery, the validated outcome scores used, and the techniques for evaluating the accuracy of recall. As a result of this, a comprehensive meta-analysis could not be performed. Additionally, this systematic review is limited by the overall quality of included studies, which were vastly non-randomized cohort studies.

## Conclusions

Recall bias plays a prominent role in orthopedic surgery, specifically, as it pertains to the patient’s recall of their preoperative symptoms/quality of life after undergoing surgical intervention. Our systematic review identified 20 studies that reported on recall bias associated with orthopedic surgery. Based on the aggregation of data from these studies, the three-month postoperative mark tends to be the time point at which patient recall transitions from being accurate to unreliable. Unsurprisingly, age appears to be an additional factor contributing to patient recall bias, with older patients demonstrating poorer recall. Given the focus of orthopedic procedures on improving pain and function, interventions to improve recall bias should be a priority for future studies. Minimizing recall bias, in addition to making the appropriate assessments in a timely manner, may allow for patients to more accurately recognize changes in preoperative symptoms after surgery.

## Appendices

**Table 4:** Databases searched and search terms used.

Electronic databases searched	Search Terms Used	Filters
PubMed, Cochrane, OVID Medline, EMBASE	(Recall Bias OR Patient Recall) AND (Orthopedic OR Orthopaedic OR Spine OR Foot OR Ankle OR Knee OR Hip OR Shoulder OR Hand OR Elbow OR Fracture OR Arthroscopic) AND (Surgery OR Procedure OR Repair)	Human, English
